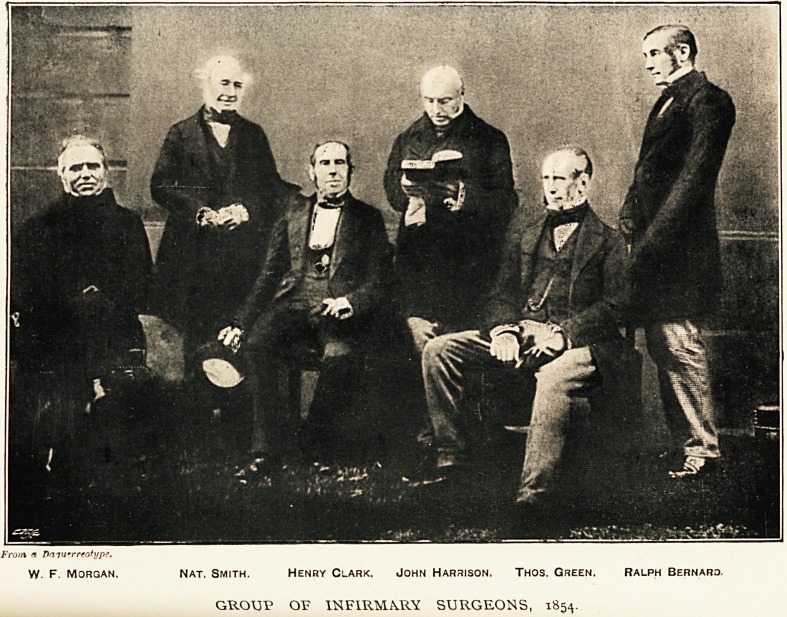# Reminiscences of the Bristol Royal Infirmary

**Published:** 1900-09

**Authors:** Arthur W. Prichard


*From a Daguerreotype.*

W. F. MORGAN. NAT. SMITH. HENRY CLARK. JOHN HARRISON. THOS. GREEN. RALPH BERNARD.

GROUP OF INFIRMARY SURGEONS, 1854.

### The Bristol Medico-Chirurgical Journal.

SEPTEMBER, 1900.

### REMINISCENCES OF THE BRISTOL ROYAL INFIRMARY.

BY

ARTHUR W. PRICHARD.

*(Extracts from the Presidential Address given before the Bath and Bristol Branch of the British Medical Association, June 27th, 1900.)*

GENTLEMEN,—You have elected me to be your president, I take it, not for any great work that I have done, nor for any excellence in any particular branch of medicine or surgery, such as some of my predecessors in the chair have achieved, but you have given me this distinction because I am senior surgeon of the Bristol Royal Infirmary, and because I happen to be a member of a family that has been represented on the staff of the Infirmary, either as consulting or acting physician or surgeon, with a very few months interval, since a year after the battle of Waterloo. It is natural, then, that I should wish to take this opportunity to draw your attention to the Infirmary, and I purpose to confine myself to a few points in the history of the Institution that have occurred during the last fifty and especially during the last twenty-five years, and to illustrate these points from the lives of some of the members of the staff who have gone from us, and whose personalities are recalled to memory by fewer and fewer among us every year.

The first noticeable event in the last half-century was the death of Mr. Lowe in 1850. Mr. Lowe had been surgeon since 1807, and it was because he and some others held office long after they could be reasonably expected to perform properly their duties to the patients and the students, that in 1843 the Committee passed a rule that no physician or surgeon should continue as such for more than twenty years. My father and Mr. Green, and Dr. Fox and Dr. Fairbrother among the physicians, were affected by that rule. In 1876 this was altered to its present reading, which is that no physician after the age of sixty, and no surgeon after the age of fifty-five, may continue upon the acting staff; but if elected to the honourable post of consulting physician or surgeon, he is requested and expected to give the valuable aid which his vast experience will have given him for the benefit of his junior colleagues in consultation.

I hold in my hand a photograph of four of the surgeons of 1854, and two consulting surgeons; the only omission is the junior surgeon but one—namely, my father, who was the photographer. I think you will agree with me that the Committee were right when they decided that it was not the best thing, at all events for the patient, that gentlemen of such mature age should be called upon to perform some critical operation at any hour of the day or night.

We ought, I think, to take more than a passing interest in these men, who did a vast amount of work for the advantage of the Infirmary, under conditions which were then very much more arduous and less pleasant than at the present time.

A few words, then, about each, not from personal acquaintance, but from facts supplied by senior friends; for though I remember them all, yet I was then too young for any opinion of mine about them to be of any value.

In 1854 the surgeons were, Mr. Harrison, Mr. Henry Clark, Mr. Thomas Green, Mr. Augustin Prichard, and Mr. Ralph Bernard. Mr. Nathaniel Smith and Mr. Morgan were consulting surgeons.

Mr. Harrison was surgeon from 1836 to 1859, and became senior surgeon in 1850 to the great advantage and comfort of the staff under him. He was a first-rate surgeon, and almost the first advocate in this country of a tonic and strengthening plan of treatment after operation. Perhaps it was his social qualities for which he will be most remembered. Painter, musician, and even poet of no mean order, his kindly and cheerful disposition, combined with a keen sense of humour, great observation, and an excellent memory, made him a delightful companion with an unflagging interest and sympathy in everything and everybody. He was a brilliant conversationalist, and once get him in the vein he would be full of anecdote, whether of his own young days, recollections of the famous riots, or of his life in the West Indies, or perhaps gruesome stories of the part he took as a young man in resurrecting bodies from the churchyards in the neighbourhood of Bristol. One who knew Mr. Harrison well and used to visit him in his latter days retains a keen recollection of his unfailing energy and invariable cheerfulness and patience under the burden of constant pain, and bodily infirmity. Never idle, in spite of his 80 years, his spirit was as bright as ever, and though his one remaining hand was much crippled by rheumatism, he would yet be touching up one of his fine sketches, or penning some graceful sonnet in his laborious writing, and all would be done with the same freshness and interest. To the last day of his life Mr. Harrison never lost the wonderful personal charm which distinguished him all through his long and gifted career.

Mr. Henry Clark was elected on the surgical staff of the Infirmary in 1843 in place of Mr. Richard Smith, who had held the surgeoncy for forty-seven years. He was one of the leading provincial surgeons, though rather a nervous operator; but it is as a teacher that his name will dwell longest in the memory of posterity. As far back as 1826 he taught anatomy in some rooms he had fitted up for the purpose close to his house in King Square. This school of anatomy was merged in the Bristol Medical School in 1854. He continued lecturing until 1860, latterly taking the course of lectures on surgery. It was owing to Mr. Clark's influence with the founder that the Suple prizes were instituted at the Infirmary. He also himself left a legacy of £500, the interest to form an exhibition for the best third year's Infirmary student at the school. His private practice was a very large one, and I think I am right in saying that he was the most prominent surgeon of his time, just as Dr. Symonds was the most prominent physician then for this part of the world. He never took a holiday—he would probably have lived longer if he had. What leisure he had he devoted to a model farm which he built at Chew Magna, and used at one time to ride out to it with one or other of his daughters in the early morning and back in time for his patients in Bristol, but he seldom slept there. He retired from the Infirmary in 1857, and died in 1861 at the comparatively early age of 59. He was one of the original members of this Association, and in 1853 president of the Branch. He was also one of the early Honorary Fellows of the Royal College of Surgeons.

Mr. Nathaniel Smith, F.R.C.S., practised in Bristol from about 1807 to 1863, when he retired to Weston. He was surgeon to the Infirmary 1816 to 1844, and was consulting surgeon at the time the photograph was taken. He is said to have been an extremely neat operator, and was most precise in his dress, so that the joke that in name and nature he was "natty" was frequently made. He was not successful in laying up for himself an abundance of this world's goods, as his unbusinesslike habits and, to a great extent, his kindness to his patients often deprived him of his just dues, although he had a vast practice in Clifton. He died in 1869, aged 87.

Mr. W. P. Morgan was born in the year 1800, passed the College and Hall in 1823, and shortly afterwards was elected house-surgeon and apothecary at the Infirmary, where he had previously been a student. He held this appointment for nine years, and was elected surgeon, in succession to Mr. William Hetling, in 1837. He was no great writer, though an able surgeon, nor did he take any active part in public matters, probably owing to the fact that he suffered from a slight impediment in his speech. He resigned the surgeoncy in 1854, and died almost suddenly of heart disease on Sunday, the 7th December, 1872. As I remember him, he was a grey-whiskered little man, neatly dressed in a long frockcoat. Documents which I have seen concerning him, and the testimonials which he received from his colleagues and the Committee, show what a very high opinion everyone had formed of his worth and character, and old friends now living tell me they look back upon Mr. Morgan as an ideal of everything that was kind and good and true.

Mr. Ralph Bernard succeeded Mr. Morgan in 1854. He opposed my father in 1850, when my father was the successful candidate. In those days the election of a surgeon to the Infirmary was a very different thing from what it is now. It somewhat resembled a parliamentary contest of the olden time: each candidate had his committee and committee-rooms; posters were stuck upon the walls, and the subscribers were brought to the Guildhall to register their votes in person, and the hourly publication of the probable numbers from the rival committee-rooms caused feeling to run very high. The students, as they were for or against any candidate, had a fine opportunity for the demonstration of their powers of persuasion. It was, I believe, owing to the general excitement and interruption of business which the 1850 election caused, that voting by proxy was introduced; and this plan was again altered in 1892 to the present system, in which a Committee of Election, composed of about fifty-seven subscribers including the House Committee and most of the staff, quietly select the candidate, and announce his appointment in the Board Room at the Infirmary. Mr. Bernard was elected in 1854, and was a surgeon of the old school rather after the type of Mr. Lowe, whose pupil he had been. He was an extremely useful member of the surgical staff, being regular and punctual, and a strict disciplinarian. He was rather a terror to the students, and the resident officers also felt his influence. His life came to a sad and abrupt end, as in 1874 he fell over a cliff in South Wales, in the presence of his wife and children, and was killed instantly.

Mr. Thomas Green, F.R.C.S., was surgeon from 1844 to 1864, and was also a surgeon of the old type,—one who, having begun his operative work in pre-anæsthetic days, had the reputation of being a quick operator, though perhaps somewhat free in the use of the knife. Born at Youghal, in County Cork, he began his medical education in Dublin, and afterwards studied in Paris at the Hotel Dieu, under Trousseau, about whom he always had something remarkable to relate. He was an Irishman to the very backbone, and possessed that tender-heartedness and generosity which is a characteristic of so many of his fellow-countrymen. This kindness and sympathy he manifested not only among his Infirmary patients, but especially towards members of his own profession in illness and distress, and many letters of gratitude from old or unsuccessful medical brethren whom he had befriended in the hour of trouble were found after his death. Some of my hearers may perhaps remember a little peculiarity he had of throwing open his necktie before taking the knife in hand to operate, a habit that was amusingly noticed by nurses and bystanders. This was probably connected with the extreme distress from irritability of the larynx which he suffered from later in life, and which nobody seemed able to relieve. As far as I know, he has been the only one of the surgeons of the Infirmary who attained the dignity of being elected an Alderman of the City of Bristol. He died in October, 1878.

Others who have passed away I shall have to mention presently, but that will be from my own recollection of them. When I entered as a three-years' dresser to my father, in 1869, the surgeons were Augustin Prichard, Ralph Bernard, Crosby Leonard, Thomas Clark, and R. W. Tibbits; and how things have changed since then! Not only have they all gone, but with them have disappeared many of the customs and formalities which had clung for generations round the office of the individuals who had risen to the high pedestal of being Surgeon to the Royal Infirmary. I am not such a *laudator temporis acti* as to wish to have the old conventionalities reintroduced. A certain amount of form and order we still observe, and they are essential for the smooth working of a great undertaking; but some of the old habits which were ably referred to two years ago by Dr. Weatherly, when he spoke in his address about his student days, have been improved away. About this time (1870) every member of the staff going round the wards kept his hat on—the only exception, I think, being Dr. Beddoe,—and the wearing of the hat was a sign that the wearer was a qualified medical practitioner. It was a privilege which we students looked forward to, and when any one of us passed the College he walked round the wards—preferably on a Saturday—with his hat on, much to his own gratification and our envy. Again, every dresser wore a dressing-gown, and the colour and cut of it showed to which surgeon that dresser belonged. As then most surgeons had two or three dressers, this was some advantage; but I am afraid that these garments were not of a very aseptic nature, and would hardly pass muster in the present age. We had to carry a pocket-case of instruments and a roll of spread ointments and plasters and lint, and, though perhaps I say it who should not, I believe that the patients were very well attended to and dressed, and notes were made of their cases, though not in the systematic way that is now in vogue.

Passing over the question of nurses, and the different social position from which most of them now come, I cannot but mention, in comparing the new with the old, the improvement there is now in the appearance of the wards. Formerly each ward was like the inside of a workhouse; now the sisters vie with each other to make their own domain the brightest, and, what with prettier colours on the walls and beds, parquetry floors, palms and plants, pictures, dressing baskets, screens and tables, couches, and other modern appliances, the place hardly seems the same.

In the beginning of 1870 the honorary staff consisted of four physicians and five surgeons, and they all were supposed to see out- and in-patients; there were two residents—the house-surgeon (Mr. Board) and the assistant house-surgeon (Mr. Ebenezer Ludlow)—and no assistant surgeon or physician. As the two residents, in addition to their ordinary daily work—attendance at operations, giving anæsthetics, and doing the work of registrar,—had to see the out-patients of any absent member of the staff, one can imagine that they were tolerably busy, and we could not grumble if they did not give us any coaching in the morning go-round. How different it is now, with five resident officers, four assistant physicians and surgeons, obstetric physician, dental surgeon, pathologist, skiagraphist, bacteriologist, every one of whom, in addition to the ordinary and in-patient staff, is ready and willing to give help to the student. I for one think that the student of the present day scarcely appreciates the advantage he has over his predecessors, and for which he ought to be thankful.

I do not mean to attempt to give a retrospect of surgery. Many such will probably be written within the next twelve months; but leaving out operations on the abdomen, which are so common now, and which in 1870 were undertaken with grave misgivings, I must mention compound fractures, amputations, and herniotomies as I remember them as a student. A case of compound fracture was always looked upon as most serious; and if it did not cost the patient his limb or his life, was one of tedious suppuration and more or less incapacity afterwards. How different now is the result with proper cleansing, drainage, perhaps wiring, and sealing the wound! Amputations were rather expected to, and generally did, heal by suppuration, and extra long flaps were made to allow for such a contingency; and as to herniotomies, the great point was not to open the sac, and I think I am right in saying that in the majority of cases the result was fatal. Hempen ligatures in all operations were used for the tying of vessels—one end cut short and the other left out at one extremity of the wound. The ligature was gradually thrown off after it had cut its way through the vessel, and if after a few days it was found by gently pulling not to be loose, a bullet or some other light weight was attached to the thread to hasten separation. Horsehair sutures from a large leash of horsehair hung up under the gallery of the operating-room were generally used; there was no systematic drainage, only what the long ends of the ligature unintentionally assisted at, and the wounds were dressed either with lint spread with ointment or with lint soaked in tincture of benzoin, which as it dried formed a hard crust over the wound. Where there was tension harelip pins and discs of cork with thread wound in a figure of 8 over them were used for button sutures, and the plan was especially useful in flap amputations.

I may also mention the decade 1870 to 1880, on account of the changes that took place then in the personnel of the staff, and so rapid were the changes that the house-surgeon of 1869

was senior surgeon ten years later. It happened thus: My father resigned in 1870, owing to the twenty years rule, although he was only fifty-one years of age, and Mr. (now Dr.) Steele was elected in his place. Mr. Bernard was accidentally killed in 1871 and Mr. Board elected. Mr. Thomas Clark retired in 1873, and Mr. Crosby Leonard and Mr. Tibbits both died in 1878, and Mr. Steele retired in the same year, so that in 1879 the five surgeons were Mr. Board, Mr. Dowson, myself, Mr. Cross, and Mr. Greig Smith, with Mr. Harsant as assistant-surgeon. This great infusion of new blood, occurring at a time when what is called Listerism was beginning and surgery was in a transition state, made a great change in everything to do with the Infirmary, and I think we take the early eighties, or what may be called the days of the Carbolic Spray, as a period in which the Infirmary was most flourishing, both as regards its popularity with the public and the number of students attending. The starting of University College had, of course, a good deal to do with this.

I will now very briefly refer to those who, by death or resignation, ceased to be active members of the staff about this time. Among the physicians, Dr. Beddoe and Dr. Brittan resigned in 1873, and Dr. Fox in 1877.

Dr. Brittan's connection with the Infirmary was one of seventeen years, and no one upheld the dignity of the office of physician with more grace than he. Many of us can recall the eloquence and grandeur of his speech in the Infirmary Museum, when he returned thanks to Lister on the occasion of his visit to us to give Bristol medical men a demonstration of the details of his method of antiseptic operating. Dr. Brittan was a man beloved by his pupils and highly esteemed by his colleagues, and a few years after his resignation of the physicianship he retired from the profession.

Dr. Fairbrother, who retired in 1876, was a man whose peculiarities and curious individualism have been referred to more than once in old Infirmary students' speeches. No one that was associated with him can forget him. He was a tall, somewhat bent, red-faced old gentleman, with a long frockcoat, the tail of which he used to take hold of to open any door and to wipe his stethoscope with; very voluble in his speech, and with a great disrespect of his h's. He was looked upon almost as a joke, but he was most interesting in his wonderful memory of the past, and to go round with Dr. Fairbrother was a great relaxation from the more scientific clinics of the other physicians. He never would commit himself to a diagnosis, and would never write a word; what he did in the out-patient room when he could not get any one of us to write for him I do not know. When I asked him for a testimonial, he agreed to give me one as a great favour if I would write it, or get it written for me, and he would sign it. Nevertheless, the patients loved him, and he was most successful in treatment, going thoroughly into the case, and (by his sympathy and appreciation of what he made them describe) getting to the bottom of their maladies; for he knew what would relieve them, and how to direct its application. If he did nothing else as regards us students, he opened for us an insight to the details of the living of many of the class that the ordinary Infirmary patient is drawn from.

Of Crosby Leonard, who died in October, 1879, at the early age of 51, I can say that personally I had for him the highest regard; and, next to my father, he was the one of all men whose opinion I sought on matters connected with the profession, whether one of surgical diagnosis or treatment, or a question of ethics; it was always willingly given, and I think he seldom made a mistake. His life was one full of work and success, and his death made a peculiar gap in the profession which to some of us was never quite filled up. He was a man whose vast experience was to the profession thrown away, for he was a most careful observer, and kept records of all his operations and unusual cases, yet his name seldom or never appeared among the contributions to the medical journals. Perhaps he meant to publish his deductions later, but his premature death deprived the medical world of a valuable and true account of the results of methods of treatment that come under the ken of a hospital surgeon with a large practice.

No one of the staff about this time did more, or rather began more, for the Infirmary than Mr. ROBERT W. TIBBITS, who was surgeon from 1868 to 1878. He was an excellent surgeon, and was a man of wonderful energy, and one who took the well-being of the Infirmary tremendously to heart. He was very outspoken, very popular with the students, but in his great push for reform not so popular with the older and more conservative members of the committee and staff. It was he who realised that the want of success that followed so many of the operations in that pre-Listeran epoch was due, not to want of skill on the part of the operator, but to the unsanitary condition of the house, and it was he who initiated and brought about the remodelling of the place which was carried out in 1875–1876. Temporary premises were taken at the bottom of Colston Street, where the work of the Infirmary was carried out during the alterations. All old flooring and woodwork, all old plaster and windows, were removed and replaced by new, and a system of drainage introduced in which the lavatories were placed in projecting turrets outside the walls and ventilated into the open air, and a proper supply of Company's water laid on instead of the old well, and a cottage was built at the bottom of the garden for erysipelas and allied diseases. This was all done in a satisfactory manner at a cost of £20,000, and the Infirmary was opened again in September, 1876, with the sanitary arrangements then perfect up to date. The result was immediate, especially in respect to surgical work; but Mr. Tibbits did not live long enough to see the fruit of his labours. He died of apoplexy in 1878, and his funeral at Westbury was attended by a larger number of friends of the Bristol Royal Infirmary than probably has ever assembled on a similar occasion.

## Figures and Tables

**Figure f1:**